# Intubation of the Larynx, with History of Cases

**Published:** 1886-03

**Authors:** F. E. Waxham

**Affiliations:** Professor of Diseases of Children, College of Physicians and Surgeons of Chicago; 3449 Indiana Ave.


### INTUBATION OF THE LARYNX, WITH HISTORY OF CASES.

*By* F. E. WAXHAM, M. D., *Professor of Diseases of Children, College of Physicians and Surgeons of Chicago.*

[Inaugural Thesis read before the Gynæcological Society of Chicago, January 15th, 1886.]

In the *Medical Record* of Feb. 21st, 1885, there appeared a brief article, by E. F. Brush, M. D., on the subject of intubation, in which he referred to two or three cases of croup treated in this manner by Dr. J. O'Dwyer, of New York. Being disheartened with tracheotomy, I rejoiced at this new departure. Although intubation of the larynx had previously been attempted in France, it was entirely abandoned, and to Dr. O'Dwyer belongs the great credit of reviving this operation, and so modifying it as to make it a practical and successful procedure. The recent literature on this subject is somewhat meager. In addition to the above reference, articles of more or less value have appeared, referring to it, in the *New York Medical Journal*, of Aug. 8th, 1885 ; the CHICAGO MEDICAL JOURNAL AND EXAMINER, of June, 1885, of November, 1885, and the *Archives of Pediatrics*, of November, 1885.

In the *New York Medical Journal*, of Aug. 8th, will be found a most interesting article on the subject by Dr. O'Dwyer, who gives a history of his investigations, extending over a period of several years, and of the various changes made in the instruments, and also illustrations of the same.

Intubation possesses many advantages over tracheotomy.

1. No opposition is met with on the part of parents and friends; quite a contrast to the difficulty with which we usually meet in obtaining the consent to tracheotomy.

2. It relieves the urgent dyspnœa as promptly and as effectually as tracheotomy, and if the child dies there is no regret that the operation was performed, and no discredit attached to the physician.

3. There is less irritation from the laryngeal tube than from the tracheal canula. As the tube is considerably smaller than the trachea, it does not press upon it firmly at any portion, excepting at the chink of the glottis.

4. Expectoration occurs more readily than through the tracheal tube.

5. As the tube terminates in the throat, the air that enters the lungs is warm and moist from its course through the upper air-passages, and there is less danger of pneumonia.

6. It is a bloodless operation.

7. It is more quickly performed, and with less danger.

8. There is no open wound that may be the source of constitutional infection.

9. Convalescence is more rapid, as there is no ghastly wound to heal by slow granulations.

10. The patient does not require the unremitting care of the physician, as in tracheotomy.

11. I believe it to be a more successful method of treating croup, either diphtheritic or membranous, than tracheotomy.

The only objection to the operation of intubation is the difficulty of performing it. This objection can be overcome by continued practice upon the cadaver. While the inexperienced will have great difficulty in entering the larynx, and will almost invariably pass into the œsophagus, yet, with sufficient practice upon the dead body, the operation may be performed quickly, easily and safely; indeed, one trial and ten seconds should be all that we should ask for. The operation of removing the tube is even more difficult than its introduction, and calls for the greatest skill and caution. In only three cases, however, have I met with difficulty. In one case the tube, which was one of the primitive ones, slipped into the trachea, but was removed, under ether, at the first attempt. In another case the child was but twenty months of age, and the tube was held so closely and tightly that it was removed with difficulty, and ether was given in this case. In another case the child was so nearly dead that no gagging occurred, and consequently the head of the tube did not rise from the vocal cords, and removal was difficult, although accomplished without ether. With these three exceptions, the tube was extracted quickly and without difficulty. These objections, although serious, and objections that will necessarily confine the operation to the hands of the most expert, are the only ones that have appeared in an experience of fourteen cases coming under my care. In regard to the comparative value of tracheotomy and intubation, we would say that as yet we have not sufficient data, but the outlook for the new operation is most encouraging, so much so that I shall never again, I am fully convinced, perform tracheotomy for membranous or diphtheritic croup. Dr. O'Dwyer, in perfecting and modifying these instruments for intubation of the larynx, has accomplished one of the greatest triumphs of modern surgery. I would beg leave to submit a report of the following cases coming under my observation. It has been my pleasure to perform this operation seventeen times, upon little patients who in every instance were in imminent danger of suffocation from either diphtheritic or membranous laryngitis. Of this number, eight have made perfect and complete recoveries. The ages varied from 16 months to 5 years. Six cases were 3 years or under, and seven cases were diphtheritics—two conditions under which tracheotomy is rarely successful. The ages of the successful cases were 5 years, 20 months, 2 years and 2 months, 5 years, 5 years, 3 years, 4 years, and 5 years, respectively. All were in imminent danger of suffocation, and in five cases tracheotomy had been proposed but declined. This is certainly a grand record for the new operation. Two questions will naturally arise in considering these statistics. First, were the cases as serious as represented? and, second, might not these cases have recovered without the operation? In answer to these queries, it may be said that in every case muco-pus and shreds of false membrane were rejected, showing that they were all cases of diphtheritic or membranous laryngitis; and, second, all, with two or three exceptions, were seen by other physicians, who invariably predicted a fatal termination, unless relieved by surgical measures. It is unnecessary to refer to the unsuccessful cases, for it may be taken for granted that they were serious ones. Of the successful cases, patient No. IV. was attended by Dr. Behrend, who sent for me to perform tracheotomy, but intubation was substituted. Dr. Behrend, as well as myself, was fully convinced that the child must soon have died without surgical aid. This patient was subsequently seen by Dr. H. T. Byford.

Case VI. was seen in consultation with Drs. Dahlberg and Appleby; both physicians were positive that danger was imminent, and that the patient must die within a few hours. Dr. Helm also witnessed the operation, and can testify to the critical condition of the patient.

Case VIII. was attended by Dr. Kossakowski, who expressed the opinion that the only hope was in an operation. The patient was also seen by Dr. Sullivan on one occasion, when it became necessary to reintroduce the tube; and on still another similar occasion by Dr. D. S. Clark, of Rockford.

Case XI. was attended by Dr. Valin, who stated that it was an impossibility for the child to live until morning without an operation,

Case XIII. was pronounced utterly hopeless by both Dr. C. E. Caldwell and Dr. Ogden.

Case XIV. was in the most critical condition, and Professor Quine stated that "any rational physician would positively have said that the child could not have lived longer than two or three hours."

I regret very much that I have no corroborative evidence in Case XVI., but I was fully convinced that there was not the least hope of recovery.

The most brilliant statistics of tracheotomy have been presented by Ranke, of Munich, who presents a series of cases, a trifle more than one-half of which recovered. Ranke recommends the early operation, and confesses that he has operated upon some cases that would have recovered without it. When we consider his statistics in the proper light they do not seem so brilliant, and correspond more nearly to our own statistics of tracheotomy. It must be remembered that by the most prompt and judicious treatment we will save one-third of our patients by medical measures, even if tracheotomy is abandoned. We may, therefore, consider that Ranke, in operating early—"as soon as the vocal and respiratory symptoms of laryngeal invasion become manifest"—operated upon one-third of his patients unnecessarily, as that proportion might have recovered without it. Subtracting one-third the number unnecessarily operated upon from one-half the number saved, and the result, one-sixth, will represent the true proportion saved. Not only this, but again, by operating early we shall operate upon a certain proportion of cases of severe spasmodic laryngitis. Often it is only by watching a case carefully from day to day that we are able to make a positive diagnosis. By operating upon these cases we will save every one of them; but ninety-nine out of every hundred would recover without the operation. It is an interesting fact that all of our most successful and brilliant tracheotomists advise the early operation.

The first five cases were reported in the CHICAGO MEDICAL JOURNAL AND EXAMINER of November, 1885.

CASE VI.—I was called November 3d to see an infant of twenty months, suffering from membranous laryngitis. From Dr. Dahlberg, the attending physician, I obtained the following history:

He was taken sick on October 29th, with slight fever, vomiting, soreness of the throat, and an occasional cough, indifference for food, but great thirst. The parents, thinking that he had taken a slight cold, gave him castor oil and hive syrup, etc., until October 31st, when Dr. Dahlberg first saw him. He found marked fever, temperature 102½° F., pulse 140; respiration accelerated, slight cough, and hoarseness of the voice. Expectoration scanty and tenacious in character. On examining the throat the mucous membrane of the fauces and tonsils were very much inflamed, and the sub-maxillary glands enlarged, but no membranous exudate could be detected. The mother, however, informed him that she had observed two white spots in the throat the day before. He diagnosed pseudo-membranous croup, and gave a mixture of quinine and tannic acid in simple syrup every three hours, alternating with a solution of chlorate of potash, tincture of iron and glycerine and a spray from an atomizer of lime water and carbolic acid.

November 1st, 9 A. M.—Temperature 101° F., pulse 140, respiration 38. Very little change. Fever less, otherwise no improvement. The hoarseness and cough more marked, and slight dyspnœa.

To facilitate the removal of muco-pus and shreds of false membrane from the throat and bronchi, he gave an expectorant mixture of muriate of ammonia, syrup of ipecacuanha and syrup of wild cherry bark, and during the night an emetic of sulphate of copper, and also every four or five hours the inhalation of slacking lime.

November 3d, 9 A. M.—Temperature 100° F., pulse 145, respiration 46. The pulse more frequent and weak; indeed, all the symptoms growing worse. Cough very much suppressed and hoarse, respiration greatly impeded, great dyspnœa, marked abdominal breathing and dilatation of alæ nasi. While in this condition I was called to perform intubation. It was evident that the child could live but a few hours unless relieved by surgical interference. Assisted by Drs. Dahlberg, Appleby and Helm, the laryngeal tube was quickly and easily introduced, requiring but a few seconds. The bridle securing the tube was removed and the result was magical, so far as the breathing was concerned, relieving the intense dyspnœa at once. After a few minutes he seemed to suffer but slightly, and in less than half an hour was sleeping quietly. At 5 o'clock the child awoke and called for food and water, and seemed to feel quite well. Temperature 100° F., pulse 140, respiration 36.

November 4th, 9 A. M.—Temperature 100½° F., pulse 136, respiration 36. 2 P. M.—Temperature, 101° F., pulse 140, respiration 36.

November 5th—Child has been sleeping nearly all night, and the appetite seems good. 9 A. M.—Temperature 100½° F., pulse 132, respiration 36. 8 P. M.—Temperature 99½, pulse 130, respiration 36. Cough seems more soft, but the respiration seems more noisy. One grain of quinine was given every three hours.

November 6th, 9 A. M.—Temperature, 99½° F., pulse 130, respiration 34. Breathing much easier and discharges considerable mucus by coughing. Slept from 10 P. M. until 3 A. M., when he awoke, called for food, and slept again until 8 A. M. 8 P. M.—Temperature 99¾° F., pulse 130, respiration 36.

November 7th, 9 A. M.—Temperature 99½° F., pulse 130, respiration 36. Has slept all night, and is breathing easily. At 2 P. M. three careful attempts were made to remove the tube, but being unsuccessful ether was given and no difficulty was experienced. The breathing for the following two hours was slightly impeded, with frequent coughing, discharging mucus and shreds of false membrane.

8 P. M.—Temperature 99½° F., pulse 134, respiration 36. Breathing easy and quite as good as with the tube. Quinine continued.

November 8th, 9 A. M.—Temperature 100° F., pulse 120, respiration 36. Has slept nearly all night. Awoke twice and inquired for food and drink.

8 P. M.—Temperature 98¾° F., pulse 120, respiration 36. Breathing not quite so easy, and less discharge of mucus in coughing. Appetite fair. Ordered an expectorant cough mixture.

November 9th, 9 A. M.—Temperature 99° F., pulse 130, respiration 40. Respiration more hurried and obstructed, indicating a proliferation of membrane.

8 P. M.—Temperature 100° F., pulse 130, respiration 42.

Breathing becoming laborious and noisy. Child very fretful. No appetite. Applied flaxseed poultice to chest and back. Gave enemas of warm milk and brandy every three hours.

November 10th, 9 A. M.—Temperature 100¾° F., pulse 130, respiration 46. Breathing laborious and exhausting. Voice and cough again becoming suppressed.

At 10 P. M. the tube was re-introduced without difficulty, when immediately the respiration became easy, and the child, after coughing up some mucus and shreds of membrane, slept quietly.

8 P. M.—Child sleeping. Temperature 99½° F., pulse 120, respiration 36.

November 11th, 9 A. M.—Temperature 99° F., pulse 120, respiration 36. Child slept well all night. Drank a cup of milk, a little beef tea and brandy; not so fretful.

8 P. M.—Temperature 101½° F., pulse 136, respiration 40. Child weaker and more feverish. Very little appetite. Continued enemata of milk and brandy every three hours.

November 12th, 9 A. M.—Temperature 98½° F., pulse 120, respiration 36. Breathing easy; little cough; appetite poor. Continued milk, quinine and brandy.

12 M.—Tube was again removed without difficulty. Coughed for a short time, expectorating mucus and shreds of false membrane.

8 P. M.—Temperature 99½° F., pulse 130, respiration 40. Has only slept one hour during the day, is very fretful, and will take but little food. Gave milk enemata with brandy every three hours, and port wine and water for drink.

November 13th, 9 A. M.—Temperature 99½° F., pulse 130, respiration 40. Slept well all night; breathing is easy, but the child very weak. Milk and brandy enemata continued.

8 P. M.—Temperature 98° F., pulse 120, respiration 36. Has slept six hours during the day. Drank a pint of milk and some chicken broth. Is better, but complains of soreness of the throat ; voice hoarse.

November 14th, 9 A. M.—Temperature 98° F., pulse 120, respiration 34. Appetite better and interested in play.

8 P. M.—Temperature 98½° F., pulse 120, respiration 34. Sleeps well and takes food better. Voice still hoarse.

November 15th, 9 A. M.—Temperature 98° F., pulse 100, respiration 30. Much improved. Appetite quite good.

November 16th, 9 A. M.—Temperature 98° F., pulse 100, respiration 30. Child decidedly better, but the voice still hoarse.

December 4th.—Child about the house and in its usual health, with the exception that the voice continues somewhat hoarse. This is the youngest patient that has yet been saved by intubation.

CASE VII.—November 9th I was called to perform intubation upon a little child four years of age, suffering from severe constitutional diphtheria with invasion of the larynx. The child had been sick for five or six days with diphtheria, and the larynx had been involved two days. The child was in imminent danger of suffocation. Ably assisted by Dr. Bosworth and Prof. Nelson, tubage was performed without the least difficulty, and with immediate relief to the urgent symptoms. The child died easily forty-eight hours later from the profound constitutional infection.

CASE VIII.—November 13th I was called by Dr. Kossakowski to operate upon a child two years and two months of age, suffering from membranous laryngitis. The child was in a desperate condition, and could have lasted but a short time. On account of the distance of the patient from me and the danger that might result if the tube was rejected, a size larger than was appropriate for its age was introduced, in order to make sure of its retention. This was worn easily and with great comfort for four days, when it was removed without difficulty. The child was unable to breathe comfortably without it, and fearing the result of the continued preſsure of the large tube upon the vocal cords a smaller one was introduced. This was done with the assistance of the father and Dr. Kossakowski very quickly, not requiring over ten seconds. Again there was the immediate relief from the alarming symptoms.

On the fifth day this tube was rejected and we were summoned in great haste. The child was again in a most critical condition. The respiration was greatly impeded, the countenance pale and anxious, dyspnœa intense and the pulse feeble and rapid. The larger tube was again reintroduced, again violent spasmodic coughing occurred, with expectoration of quantities of ropy mucus and shreds of false membrane. The silk bridle was removed, and again all the alarming symptoms subsided. On the ninth day the tube was easily and quickly removed, and although the respiration was somewhat embarrassed it was thought best not to reintroduce the tube. In about three hours we were again summoned in great haste, and on reaching the child it was found nearly asphyxiated. The lips and fingers were blue, the face livid, and the extremities cold. Respiration was carried on with the greatest difficulty. The child would soon have been dead. The tube was quickly reintroduced, small pieces of membrane rejected, and again the little patient was saved from impending death.

The child took abundance of nourishment, and wore the tube without annoyance. On the thirteenth day, assisted by

Dr. Kossakowski, of this city, and Dr. D. S. Clark, of Rockford, the tube was again removed without difficulty. Respiration considerably embarrassed without the tube. An emetic was given which acted promptly, but only increased the difficulty of respiration. After waiting one hour and a half, during which time the dyspnœa gradually increased, it was considered unsafe to leave the child, and the smaller tube was again reintroduced. On the fourteenth day this tube was rejected, but it was not necessary to reintroduce it. The child made a rapid and satisfactory convalescence.

CASE IX.—Nov. 16th, Dr. Kossakowski again called upon me to perform intubation upon a little child three years old. It was a most unfavorable case. The family were crowded in two small rooms, and the child was found lying on a feather mattress on the floor in the corner of the kitchen. It was in the last stages of suffocation from membranous croup. One of the neighbors held the child in the lap, and with the assistance of the doctor intubation was quickly performed. The child revived and the next day took dinner with the family. The child died forty-eight hours later from extension of the exudation below the tube.

CASE X.—Nov. 20th I was requested by Professors Cassleberry and Hatfield to see a boy of five years in the very last stages of membranous croup. The patient had been sick about eight days, and twice there had been reformation of the membrane after its complete rejection. At this time there was evidence of extensive exudation into the bronchi. Both physicians pronounced the case a most unfavorable one, and it was scarcely expected that intubation would give relief. However, the operation was quickly performed and large quantities of mucopus and softened membrane expelled through the tube. Contrary to our expectation, great relief was given. The patient died about fifty hours after the operation with evidence of reaccumulation of membrane in the bronchial tubes together with pneumonia of the left lung. In this case considerable difficulty was experienced in removing the tube; several attempts were necessary, but it was extracted without ether. The child was so near death that an anæsthetic was not admissible, and it was hoped that by removing the tube the respiration might improve, but the unfavorable symptoms were even more distressing, and death occurred two hours later.

CASE XI.—Nov. 21st I was called to see Charley B., a boy of five years, a patient of Dr. Valin, who kindly gave me the following history: He was taken ill with a severe form of pharyngeal diphtheria, Nov. 7th. Both tonsils and uvula were covered with diphtheritic membrane. The temperature for several days remained at 102½° F., and the pulse 130. On the 18th the membrane disappeared from the fauces. On the 19th there was evidence of extension of the disease into the larynx. On the 21st the child was in imminent danger of suffocation. The dyspnœa was intense, and the child was struggling desperately for breath. He was covered with perspiration, and the features were livid and anxious, and the pulse was becoming weak and very rapid. The child certainly could have lasted but a few hours longer. With the aid of the father and Dr. Valin intubation was performed, requiring but a few seconds. There immediately followed severe spasmodic coughing, with expectoration of quantities of muco-purulent matter and shreds of membrane. When the silk bridle was removed the respiration became easy, the cough subsided and the child passed into a quiet natural sleep.

Temperature, 102° F.; pulse, 120; respiratinn, 20.

Nov. 22nd, 4:30 A. M., temperature, 102° F.; pulse, 120; respiration, 24. 8 A. M., temperature, 98½° F.; pulse, 100; respiration, 22.

Nov. 23rd, at 4 A. M., the tube was expelled during a violent attack of coughing. The dyspnœa at once returned, and when seen, the child was again laboring desperately for breath. The tube was reintroduced, and again all violent symptoms subsided. At 9 A. M., temperature, 100½° F.; pulse, 120; respiration, 22.

Nov. 24th, temperature, 100½° F.; pulse, 120; respiration, 22.

Nov. 25th, temperature, 100° F.; pulse, 120; respiration, 24.

Nov. 26th, at 4 A. M., the tube was again expelled, this being the fifth day after its first introduction. As the respiration was comparatively easy the tube was not reintroduced. Temperature, 98½° F.; pulse, 126; respiratinn, 24.

Nov. 27th, temperature, 100° F.; pulse, 120; respiration, 24.

Nov. 29th, temperature, 98½° F.; pulse, 108; respiration, 22.

Dec. 5th, child entirely well, and but very slight hoarseness of the voice.

CASE XII.—Nov. 30th, Prof. Holroyd called me to perform intubation on a little boy of four years. The child had been sick for several days with diphtheritic croup, and when seen was failing rapidly. The tube was quickly introduced with the usual relief. About 1 A. M., Dec. 1st, a portion of membrane, about 1½ inches in length by ½ in. in width, became detached and completely occluded the tube. As a result, both the tube and membrane were rejected. We were at once summoned, but before we were able to reach the child he was nearly asphyxiated. The tube was again introduced with prompt and great relief. The respiration was considerably accelerated but easy, and the child was left sleeping. After giving explicit directions in regard to the use of the instruments they were left with the doctor. About 4 A. M. the tube was again rejected. We were at once summoned, but before arriving Professor Holroyd had succeeded admirably in introducing the tube. The respiration, although less easy, was quite rapid, the skin hot and the pulse very rapid. There were evident symptoms of congestion of the lungs. A hot mustard foot-bath was given, warm flaxseed poultices covered with oiled silk applied to the chest and four-grain doses of quinine given, alternating with 1-32 gr. of bi-chl. mercury. The child died at 4 P. M., very easily, evidently from congestion of the lungs.

CASE XIII.—Through the courtesy of Dr. C. E. Caldwell, I was called, Dec. 2nd, to see Mark H., a boy of five years. The doctor has kindly given me the following history: The boy had been sick one week with the measles when, as the eruption was fading, diphtheria occurred. The pharynx was well covered with the diphtheritic deposit, which rapidly extended into the larynx. Dec. 2nd, the child was in great danger of suffocation, and certainly could have lived but a few hours. With the assistance of Dr. Caldwell and Dr. E. J. Ogden, the operation of tubing was quickly accomplished. Large quantities of mucuo-pus and broken down membrane were expelled and the patient was soon sleeping quietly. 1-32 of a gr. of bi-chl. mercury had been given every half hour and was continued, which constituted the only treatment.

6 P. M. Pulse, 150; temperature, 102° F.; respiration, 30. 10 P. M. Pulse, 150; temperature, 101° F.; respiration, 24. Takes nourishment well.

Dec. 3rd, 9 A. M. Pulse, 140; temperature, 100° F.; respiration, 24. Rests well and takes nourishment in liberal quantities. 9 P. M. Pulse, 150; temperature, 99° F.; respiration, 24.

Dec. 4th, 9 A. M. Pulse, 136; temperature, 99° F.; respiration, 24. 9 P. M. Pulse, 130; temperature, 98½° F.; respiration, 24.

Dec. 5th. The child was seized with violent coughing and vomiting and the tube was rejected. Pulse, 120; temperature, 98½° F.; respiration, 24.

Takes nourishment plentifully. Voice quite hoarse.

Dec. 6th. Continues to improve.

Dec. 7th. Is convalescent.

CASE XIV.—Dec. 3d I was informed by Dr. Dahlberg that he would send a little patient, suffering from membranous laryngitis, to the hospital of the College of Physicians and Surgeons, for me to perform intubation upon. The child, four years of age, had been suffering for three days, and the doctor thought, as the surroundings were most unfavorable, that his chances would be far better at the hospital than at home. The people were advised to take the child in a closed carriage, but feeling too poor to afford it, the little patient was taken in a street car, a distance of several miles. The child arrived in a most critical condition, and those who witnessed the operation certainly will testify that the patient could not possibly have survived more than a very few hours. Assisted by Dr. Dahlberg and Prof. Quine, the operation was performed in the presence of the class. The tube when introduced was entirely filled and occluded with false membrane. After struggling for a moment the tube was rejected, a fortunate occurrence, as the child could not have lived with the tube so completely occluded. As this tube was passed with some difficulty, a size smaller was quickly introduced and all alarming symptoms subsided.

Dec. 4th, 9 A. M., temperature, 100° F.; pulse, 156; respiration, 36. 3 P. M., temperature, 100¾° F.; pulse, 137; respiration, 26.

Dec. 5th, 9 A. M., temperature, 101½° F.; pulse, 150; respiration, 22. 5 P. M., temperature, 100¾° F.; pulse, 142; respiration, 26.

Dec. 6th. The inflammation had extended upward into the nasal passages and the left nostril was filled with a bloody muco-purulent secretion. 9 A. M., temperature, 100° F.; pulse, 150; respiration, 32. 3 P. M., temperature, 101½° F.; pulse, 155; respiration, 40.

Dec. 7th, 8 A. M., temperature, 101½° F.; pulse, 140; respiration, 36. 1 P. M., temperature, 102° F.; pulse, 150; respiration, 48.

At 6 P. M., assisted by Prof. Earle, Dr. Dahlberg and Mr. Anderson, the tube was quickly and easily extracted, after remaining in position a little over four days.

The patient remained very feeble for the next few days, but gradually the strength returned, and the child was discharged from the hospital Dec. 14th entirely out of danger. In this case the treatment consisted of 1-32 gr. of bi-chloride of mercury for the first two days, given every hour; for the subsequent two days less frequently, and after the removal of the tube, full doses of iron and quinine.

CASE XV.—Dec. 9th I was called to see a little boy of four years, and learned that he had been sick for nearly a week. The child had sore throat, white spots on the tonsils and soft palate, and considerable fever. Two days before, the cough became croupy and the respiration embarrassed, and the father said that during the previous night he thought the child would choke to death every minute. The child had been under domestic treatment. Upon inspecting the throat, ulcerations were observed upon the uvula and both tonsils, covered by a thin coating of membrane. The child was in great danger, the voice and cough suppressed, deep sinking in of the tissues at the base of the thorax, pale and covered with perspiration. As the symptoms admitted of no delay and as no physician was at hand, the mother courageously held the child in her lap, while the father held the head, the gag placed in position, and the tube quickly introduced, requiring about ten seconds. Acting upon a suggestion of Dr. O'Dwyer, a salve containing 25 per cent. of nitrate of silver was thoroughly coated over the tube just below the head. The action of the astringent upon the false membrane and the tissues about the vocal cords, I am convinced was beneficial, although considerable irritation was produced. There is one objection to the use of nitrate of silver in this manner. It destroys the gold plating of the tube. The other astringents will perhaps serve as useful a purpose without this objection.

Just before the tube was introduced the pulse was 140; respiration, 30; temperature, 101° F. At 7 P. M., pulse, 140; respiration, 40.

Dec. 10th, 10 A. M., pulse, 140; respiration, 40. 9 P. M., pulse, 140; respiration, 48; temperature, 102° F.

Dec. 11th, 9 A. M., pulse, 130; respiration, 36; temperature, 100½° F. 9 P. M., pulse, 120; respiration, 32.

On the 12th, three days after intubation was performed, the tube was removed without difficulty. At this time as well as when the tube was first introduced considerable muco-pus and shreds of false membrane were rejected. It became unnecessary to reintroduce the tube and the child made a rapid and complete recovery.

CASE XVI.—Dec. 11th I was called by Dr. Pierpoint, of Englewood, to perform intubation upon a little patient three years old. The child had been suffering several days with a bad form of diphtheria which finally invaded the larynx. When intubation was performed the child was in imminent danger of strangulation.

The operation gave entire relief, but the patient died forty-eight hours later from the occurrence of pneumonia and from the diphtheritic infection.

CASE XVII.—Jan. 4th. Through the courtesy of Prof. Quine and Dr. Willard I was called to see a little girl five years old in the last stages of membranous croup. It was evident to all that the child must have died in a very short time. Professor Quine was positive that the patient could not have lived until midnight. When the tube was introduced a large piece of membrane was rejected and all the alarming symptoms subsided. The next day the child was up and playing about the room nearly all day. The little patient experienced no inconvenience from the presence of the tube excepting when she swallowed liquids. Every day she was about the room, and on one occasion even helped her mother to wipe the dinner dishes. On the third day the tube was removed with the assistance of Dr. Willard, of this city, and Dr. Kimball, of Rockford. There was no necessity of reintroducing the tube, and the child made a rapid recovery. This had been the most remarkable case that has yet come under my observation.

Limited as is this experience, I feel that it is sufficient to establish this as a legitimate operation, and one that is destined to entirely supersede tracheotomy.

3449 Indiana Ave.

